# Supplementary material for: Reconstructing Mayotte 2018–19 Rift Valley Fever outbreak in humans by combining serological and surveillance data
Source: Commun Med (Lond). 2022 Dec 21;2:163. doi: 10.1038/s43856-022-00230-4 (PMC9772320; doi:10.1038/s43856-022-00230-4)
Supplement: Supplementary file 2 — Supplementary Information [file 43856_2022_230_MOESM2_ESM.pdf]

Supplementary information

Reconstructing Mayotte 2018-19 Rift Valley Fever outbreak in humans by combining serological and surveillance data

Jonathan Bastard, Guillaume André Durand, Fanny Parenton, Youssouf Hassani, Laure Dommergues, Juliette Paireau, Nathanaël Hozé, Marc Ruello, Gilda Grard, Raphaëlle Métras, Harold Noël

Supplementary Note 1. Geographical areas and demography data

Using data from Mayotte island, a previous publication [1] performed network analyses to identify clusters of communes that had similar livestock movement patterns. The authors found that communes located in the central region of this island (Central communes) tended to be classified as one cluster, while communes located in its outer region (Outer communes) tended to be classified as another. Central communes were shown to act as a hub in the livestock movement network, with a denser connectivity between Central communes and from Central to Outer Communes, than from Outer to Central communes and between Outer communes. [1] and [2] demonstrated that these livestock movement patterns in Mayotte affect the spread of RVFV in livestock populations and, ultimately, to humans. This justifies using this classification of communes to study the extent of the 2018-19 RVF outbreak in humans.

The "Central communes" and "Outer communes" geographical areas are depicted in Figure 1 (main text). Central communes include Sada, Tsingoni, Ouangani, Dembeni and Mamoudzou, and were populated by 122,578 inhabitants in 2017, including 68,189 over 15 years old. Outer communes include Chirongui, Kani-Keli, Bandraboua, Pamandzi, Chiconi, Boueni, Bandrele, Dzaoudzi, Mtsamboro, M'tsangamouji, Acoua and Koungou, and were populated by 133,940 inhabitants in 2017, including 76,073 over 15 years old. The total population of Mayotte Island was 256,518 in 2017, including 144,262 over 15 years old [3].

Supplementary Table S1. Estimated parameters of the model

Supplementary Table S1. Estimated parameters of the model: description, unit and prior distribution. As described in Table 1 (main text), N_i is the size of subpopulation i.

Parameter	Description	Unit	Prior distribution
τ	Reporting fraction	-	Uniform: [0,1]
$S_{0,i}$	IgG seroprevalence in subpopulation i (in people over 15 years old) before the outbreak	-	Uniform: [0,1]
D	Time between infection and IgG detectability	Weeks	Categories of equal probabilities: {0,1,2,...,7}
$p_{1,i}$	Total number of people over 15 years old in subpopulation i infected during the outbreak (parameter of F_i lognormal distribution)	-	Exponential transformation of uniform: [log(50),log(N_i)]
$p_{2,i}$	Parameter of F_i lognormal distribution	-	Uniform: [$p_{3,i}$+log(10), $p_{3,i}$+log(30)]
$p_{3,i}$	Parameter of F_i lognormal distribution	-	Uniform: [0,1]

Supplementary Figure S1. Distribution of the serological data

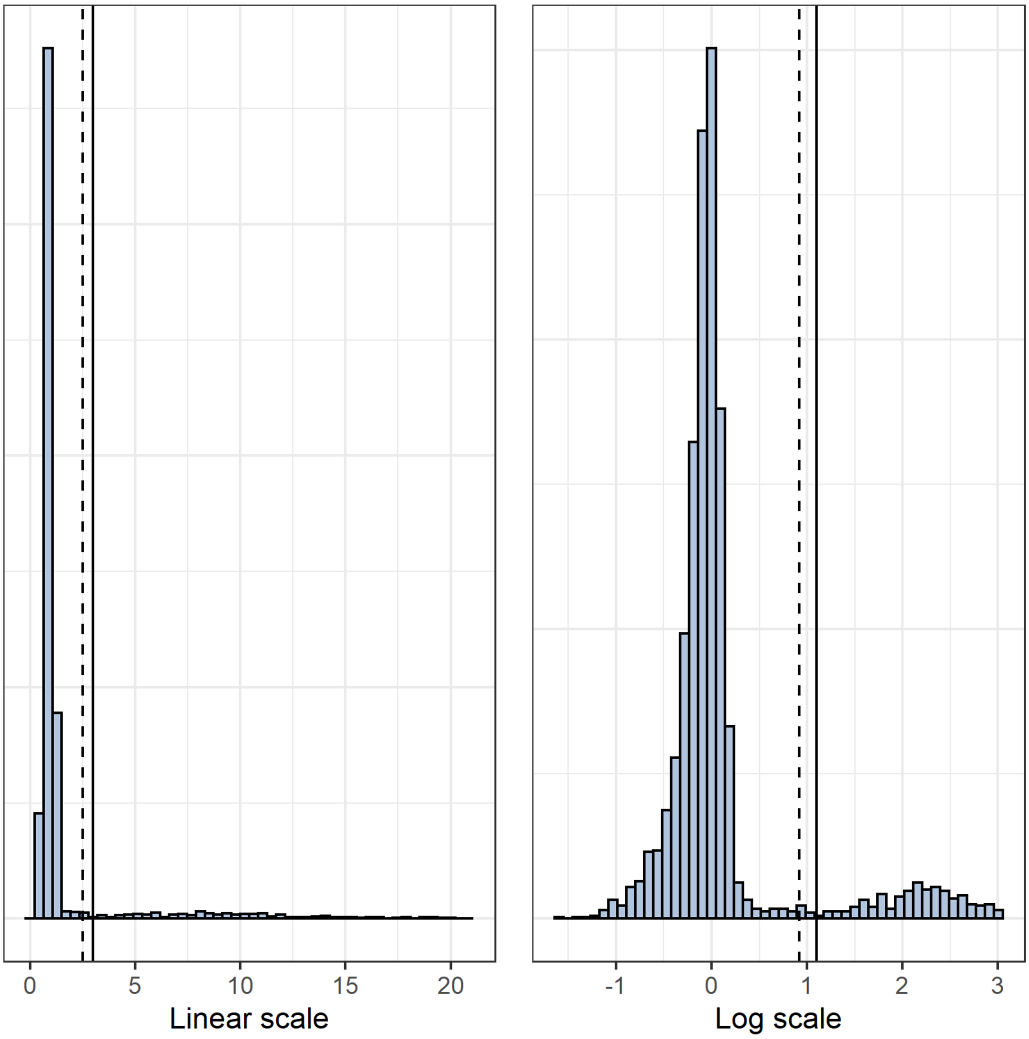

Supplementary Figure S1. Distribution of the ratios of optical density (in linear scale, left, and in log scale, right) between wells coated with inactivated virus and wells coated with negative antigen, obtained from the 2,853 blood samples collected as part of the seroprevalence study in Mayotte island. The solid line represents the cut-off of 3 considered in the main analysis to classify a sample as positive (right) or negative (left) to RVFV IgG antibodies. The dashed line represents the cut-off of 2.5 used in the sensitivity analysis.

Supplementary Table S2. Parameters estimates

Figure 2 (main text) shows the prior and posterior distributions of estimated parameters, and Supplementary Table S2 describes their posterior median and 95% credible interval (highest posterior density interval).

Supplementary Table S2. Model's parameters estimates in the main analysis: median of the posterior distribution and 95% credible interval (highest posterior density interval).

Parameter	Unit	Estimate (median and 95% credible interval)
τ	-	0.012 (0.0067 - 0.022)
$S_{0,Central}$	-	7.2% (4.9% - 9.7%)
$S_{0,Outer}$	-	4.0% (1.8% - 6.4%)
D	Weeks	1 (0 - 4)
$p_{1,Central}$	-	4,618 (2,186 - 7,409)
$p_{2,Central}$	-	2.96 (2.89 - 3.03)
$p_{3,Central}$	-	0.079 (0.062 - 0.108)
$p_{1,Outer}$	-	6,130 (2,579 - 9,265)
$p_{2,Outer}$	-	3.02 (2.96 - 3.08)
$p_{3,Outer}$	-	0.074 (0.052 - 0.101)

Supplementary Note 2. Sensitivity analysis – Unstratified data

In the main analysis, the data were stratified into two subpopulations living in two distinct geographical areas: Central and Outer communes of the island. Here, to test the robustness of the analysis to this stratification, we present the model's fit (Supplementary Figure S2) and parameters estimates (Supplementary Table S3) when the data on these two populations are not stratified.

Supplementary Table S3. Model's parameters estimates in the sensitivity analysis using unstratified data: median of the posterior distribution and 95% credible interval (highest posterior density interval).

Parameter	Unit	Estimate (median and 95% credible interval)
τ	-	0.013 (0.0070 - 0.024)
$S_{0,Mayotte}$	-	5.7% (3.7% - 8.0%)
D	Weeks	1 (0 - 4)
$p_{1,Mayotte}$	-	10,998 (4,971 - 16,473)
$p_{2,Mayotte}$	-	3.00 (2.95 - 3.04)
$p_{3,Mayotte}$	-	0.073 (0.063 - 0.090)

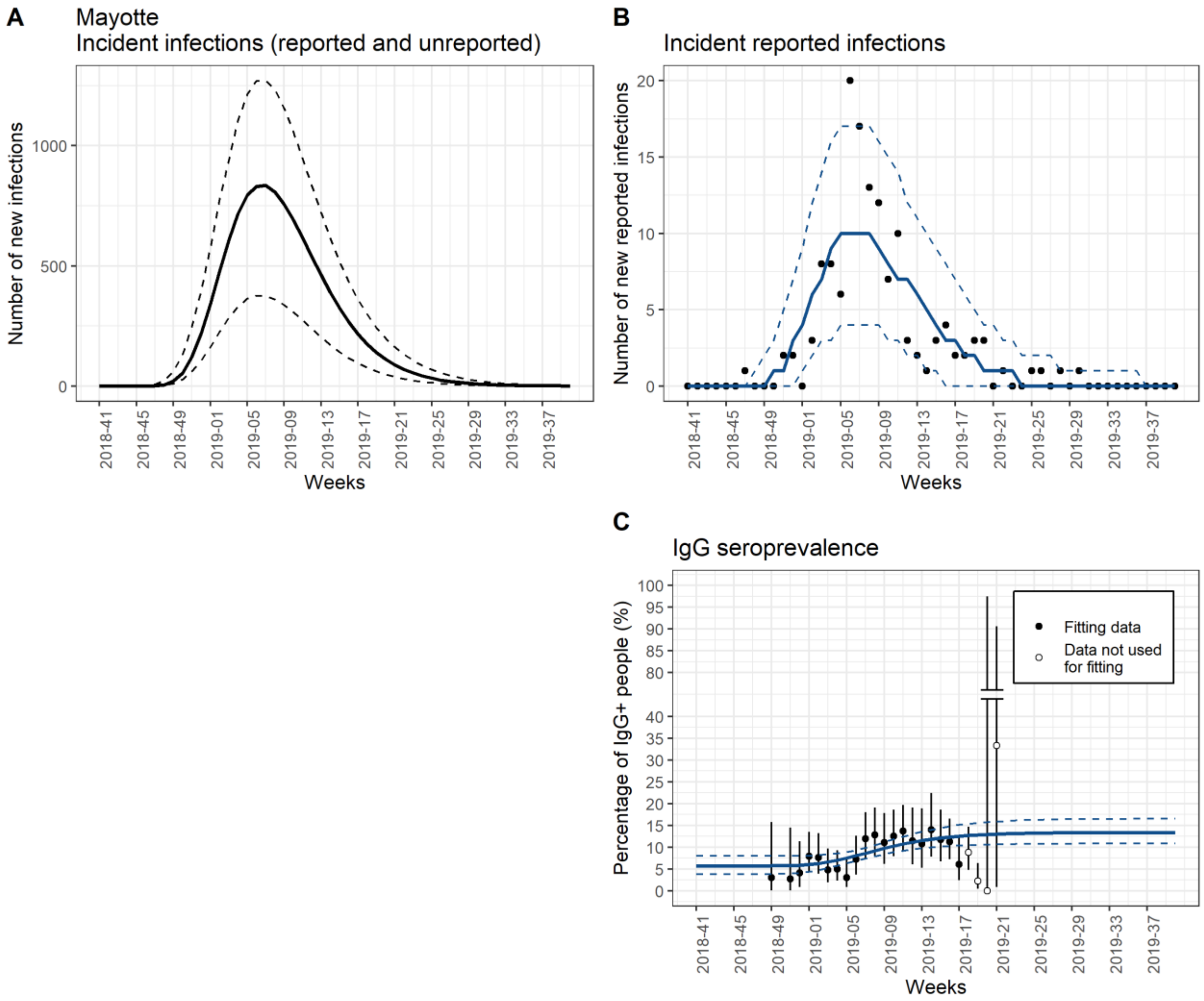

Supplementary Figure S2. In the sensitivity analysis using unstratified data: predicted number of weekly incident human infections, reported or unreported to the surveillance system (panel A), number of incident human infections reported to the surveillance system (panel B), and IgG seroprevalence in humans (panel C), in the population over 15 years old. Lines (solid and dashed) are model predictions (median and 95% prediction interval respectively, 5000 repetitions of the model). Dots are observed data (number of reported cases in panel B, and proportion of IgG seropositive tests in panel C). Vertical bars (in panel C) are 95% confidence interval for the proportion (Clopper-Pearson method). Serological data from week 2019-18 (early May 2019) onwards were not used for model fitting, because the representativeness of the sampled population was compromised (see Methods). In panel C, for visualization purposes, the Y-axis is cut between 40 and 80.

Supplementary Note 3. Sensitivity analysis – Different serological cut-off

In the main analysis, we considered that samples whose OD ratio was > 3 were IgG positive. To test the sensitivity of our results to this assumption, we performed the analysis considering a cut-off of 2.5 instead of 3. With this cut-off, 264 out of 2,854 samples were classified as RVFV IgG positive. Supplementary Table S4 presents the parameters estimates in this case, and Supplementary Figure S3 shows the data and model's predictions.

Supplementary Table S4. Model's parameters estimates in the sensitivity analysis using a serological cut-off of 2.5 instead of 3: median of the posterior distribution and 95% credible interval (highest posterior density interval).

Parameter	Unit	Estimate (median and 95% credible interval)
τ	-	0.012 (0.0066 - 0.022)
$S_{0,Central}$	-	7.4% (5.0% - 9.9%)
$S_{0,Outer}$	-	4.0% (1.8% - 6.6%)
D	Weeks	1 (0 - 4)
$p_{1,Central}$	-	4,726 (2,261 - 7,491)
$p_{2,Central}$	-	2.96 (2.89 - 3.04)
$p_{3,Central}$	-	0.079 (0.062 - 0.108)
$p_{1,Outer}$	-	6,332 (3,016 - 9,770)
$p_{2,Outer}$	-	3.02 (2.96 - 3.08)
$p_{3,Outer}$	-	0.074 (0.052 - 0.102)

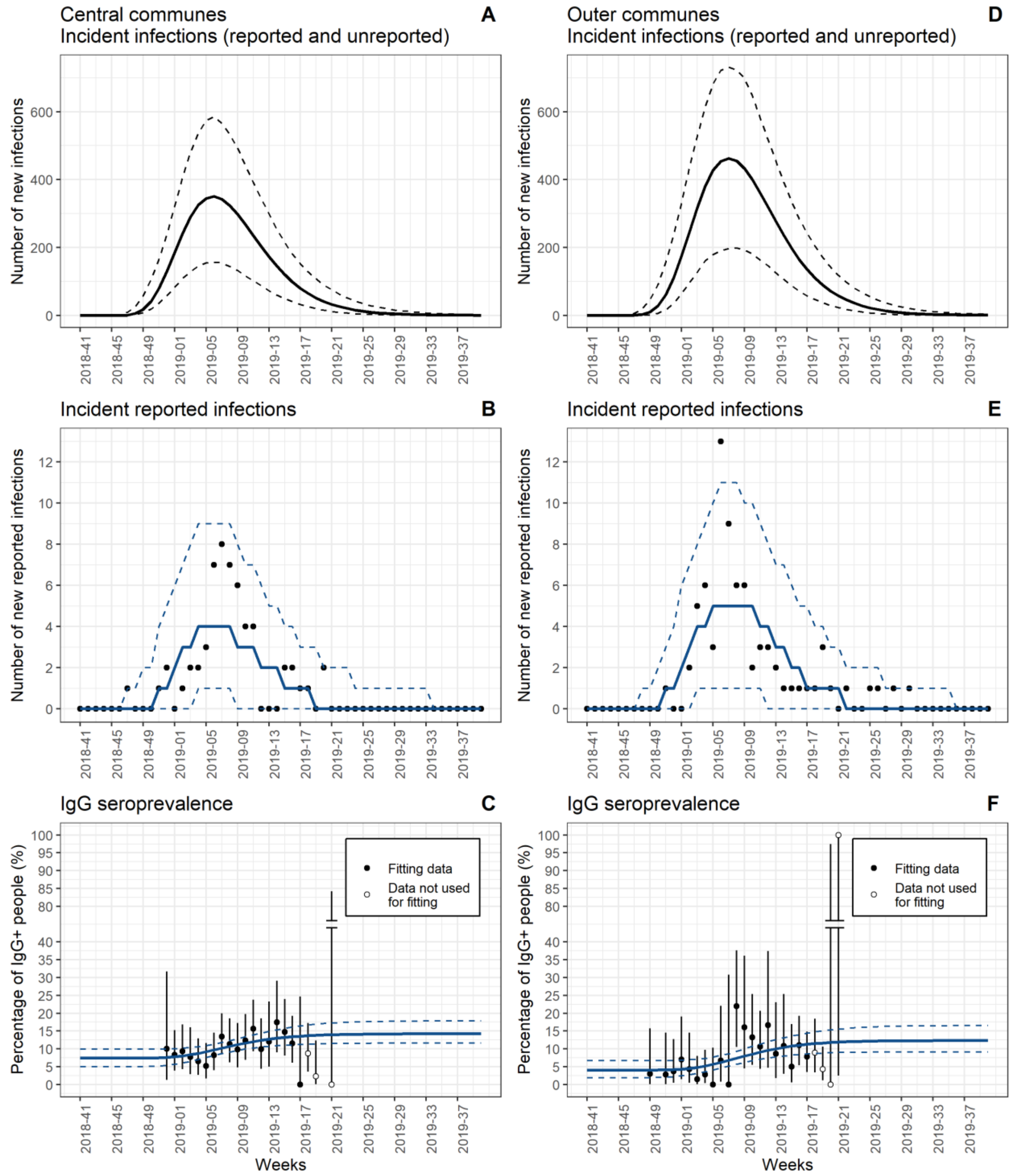

Supplementary Figure S3. In the sensitivity analysis using a serological cut-off of 2.5: predicted number of weekly incident human infections, reported or unreported to the surveillance system (A, D), number of incident human infections reported to the surveillance system (B, E), and IgG seroprevalence in humans (C, F), in the population over 15 years old. Panels A to C represent Central Communes while panels D to F represent Outer Communes of Mayotte. Lines (solid and dashed) are model predictions (median and 95% prediction interval respectively, 5000 repetitions of the model). Dots are observed data (number of reported cases (B, E) and proportion of IgG seropositive tests (C, F)). Vertical bars (in panels C and F) are 95% confidence interval for the proportion (Clopper-Pearson method). Serological data from week 2019-18 (early May 2019) onwards were not used for model fitting, because the representativeness of the sampled population was compromised (see Methods). In panels C and F, for visualization purposes, the Y-axis is cut between 40 and 80.

Bibliography of the Supplementary information

1. Kim, Y. *et al.* Livestock trade network: potential for disease transmission and implications for risk-based surveillance on the island of Mayotte. *Sci. Rep.* **8**, 11550 (2018).
2. Kim, Y. *et al.* The role of livestock movements in the spread of Rift Valley fever virus in animals and humans in Mayotte, 2018–19. *PLoS Negl. Trop. Dis.* **15**, e0009202 (2021).
3. INSEE. Recensement de la population de Mayotte en 2017. https://www.insee.fr/fr/statistiques/3284395?sommaire=4199393 (2019).
