# Supplementary material for: Reconstructing Mayotte 2018–19 Rift Valley Fever outbreak in humans by combining serological and surveillance data
Source: Commun Med (Lond). 2022 Dec 21;2:163. doi: 10.1038/s43856-022-00230-4 (PMC9772320; doi:10.1038/s43856-022-00230-4)
Supplement: Supplementary file 3 — Description of Additional Supplementary Files [file 43856_2022_230_MOESM3_ESM.pdf]

Description of Additional Supplementary Files

File Name: Supplementary Data 1

Description: Number of reported cases over 15 years old, number of people sampled as part of the seroprevalence survey, and number of RVFV IgG positive people, by week and by geographical area (i.e. by subpopulation). For reported cases, we considered the week of symptoms onset or, if missing, the week of RT-PCR confirmation. For the seroprevalence study, we considered the week of sampling.
