# Supplementary material for: Reconstructing Mayotte 2018–19 Rift Valley Fever outbreak in humans by combining serological and surveillance data
Source: Commun Med (Lond). 2022 Dec 21;2:163. doi: 10.1038/s43856-022-00230-4 (PMC9772320; doi:10.1038/s43856-022-00230-4)
Supplement: Supplementary file 5 — Reporting Summary [file 43856_2022_230_MOESM5_ESM.pdf]

nature research

Corresponding author(s): Jonathan Bastard

Last updated by author(s): Nov 21, 2022

Reporting Summary

Nature Research wishes to improve the reproducibility of the work that we publish. This form provides structure for consistency and transparency in reporting. For further information on Nature Research policies, see our Editorial Policies and the Editorial Policy Checklist.

Statistics

For all statistical analyses, confirm that the following items are present in the figure legend, table legend, main text, or Methods section.

n/a	Confirmed	
☐	☒	The exact sample size (n) for each experimental group/condition, given as a discrete number and unit of measurement
☐	☒	A statement on whether measurements were taken from distinct samples or whether the same sample was measured repeatedly
☐	☒	The statistical test(s) used AND whether they are one- or two-sided<br>*Only common tests should be described solely by name; describe more complex techniques in the Methods section.*
☐	☒	A description of all covariates tested
☐	☒	A description of any assumptions or corrections, such as tests of normality and adjustment for multiple comparisons
☐	☒	A full description of the statistical parameters including central tendency (e.g. means) or other basic estimates (e.g. regression coefficient) AND variation (e.g. standard deviation) or associated estimates of uncertainty (e.g. confidence intervals)
☒	☐	For null hypothesis testing, the test statistic (e.g. F, t, r) with confidence intervals, effect sizes, degrees of freedom and P value noted<br>*Give P values as exact values whenever suitable.*
☐	☒	For Bayesian analysis, information on the choice of priors and Markov chain Monte Carlo settings
☒	☐	For hierarchical and complex designs, identification of the appropriate level for tests and full reporting of outcomes
☒	☐	Estimates of effect sizes (e.g. Cohen's d, Pearson's r), indicating how they were calculated

Our web collection on statistics for biologists contains articles on many of the points above.

Software and code

Policy information about availability of computer code

Data collection	No code was used to collect data.
Data analysis	All analyses were performed using R version 4.2.0. Codes are available here: https://github.com/JonathanBas/RVF_Mayotte.

For manuscripts utilizing custom algorithms or software that are central to the research but not yet described in published literature, software must be made available to editors and reviewers. We strongly encourage code deposition in a community repository (e.g. GitHub). See the Nature Research guidelines for submitting code & software for further information.

Data

Policy information about availability of data

All manuscripts must include a data availability statement. This statement should provide the following information, where applicable:

- Accession codes, unique identifiers, or web links for publicly available datasets
- A list of figures that have associated raw data
- A description of any restrictions on data availability

The data supporting the findings of this study are available within the paper and its supplementary material file (SM1, Table S1).

Field-specific reporting

Please select the one below that is the best fit for your research. If you are not sure, read the appropriate sections before making your selection.

☒ Life sciences ☐ Behavioural & social sciences ☐ Ecological, evolutionary & environmental sciences

For a reference copy of the document with all sections, see nature.com/documents/nr-reporting-summary-flat.pdf

Life sciences study design

All studies must disclose on these points even when the disclosure is negative.

Sample size	In the serological study, sample size was determined according to the expected prevalence of diseases of interest [21].
Data exclusions	Due to the starting of the Ramadan, the acceptance of blood sampling as part of the Unono Wa Maore survey decreased from late April 2019 onwards, compromising the representativeness of the sampled population. As a result, we analyzed in this paper the serological data collected prior to week 2019-18, as indicated in the main text.
Replication	Model simulations were repeated 5000 times, as indicated in the main text.
Randomization	Our study did not involve allocation of individuals into experimental groups. However, in the serological study, individuals were randomly sampled in order to ensure representativeness [21].
Blinding	Our study did not involve allocation of individuals into experimental groups. Therefore, blinding is not applicable to our study.

Reporting for specific materials, systems and methods

We require information from authors about some types of materials, experimental systems and methods used in many studies. Here, indicate whether each material, system or method listed is relevant to your study. If you are not sure if a list item applies to your research, read the appropriate section before selecting a response.

Materials & experimental systems

n/a	Involved in the study	
☐	☒	Antibodies
☒	☐	Eukaryotic cell lines
☒	☐	Palaeontology and archaeology
☒	☐	Animals and other organisms
☐	☒	Human research participants
☒	☐	Clinical data
☒	☐	Dual use research of concern

Methods

n/a	Involved in the study	
☒	☐	ChIP-seq
☒	☐	Flow cytometry
☒	☐	MRI-based neuroimaging

Antibodies

Antibodies used	A homemade indirect ELISA was performed, using whole inactivated virus (Tchad 2001) and a goat anti-human IgG conjugated with peroxidase (Jackson Immuno Research, UK), as described in previous publications [26, 27].
Validation	The anti-human IgG was used and conserved as recommended by manufacturer. All plates contained positive control (from previous positive sera of our biobank).

Human research participants

Policy information about studies involving human research participants

Population characteristics	Our study focused on people living in Mayotte island, aged over 15 years old, stratified according to their place of residence (in Central VS Outer communes), as described in the main text.
Recruitment	Reported cases data were collected as part of the routine surveillance described in the main text. Participants in the serological study were recruited randomly and were representative of the population of Mayotte [21].
Ethics oversight	Unono Wa Maore research protocol was validated by the Committee for the Protection of Persons (CPP, no. 2017-A02782-51), the French ethical committee for biomedical research, and complied with MR001 reference methodology (agreement from the National Commission for Informatics and Freedoms of 25 September 2018, no. 918233). Information on the survey objectives and consent forms were read with the participants. A written informed consent was obtained from all participants that were over 15 years old. All methods were carried out in accordance with relevant guidelines and the

Declaration of Helsinki. All samples and data were anonymized at the time of collection. Therefore, sample testing and data analysis were conducted anonymously.

Note that full information on the approval of the study protocol must also be provided in the manuscript.
